# The Wnt/β-Catenin/LEF1 Pathway Promotes Cell Proliferation at Least in Part Through Direct Upregulation of miR-17-92 Cluster

**DOI:** 10.3389/fgene.2019.00525

**Published:** 2019-05-29

**Authors:** Fang Mu, Jiaxin Huang, Tianyu Xing, Yang Jing, Tingting Cui, Yaqi Guo, Xiaohong Yan, Hui Li, Ning Wang

**Affiliations:** ^1^Key Laboratory of Chicken Genetics and Breeding, Ministry of Agriculture and Rural Affairs, Harbin, China; ^2^Key Laboratory of Animal Genetics, Breeding and Reproduction, Education Department of Heilongjiang Province, Harbin, China; ^3^College of Animal Science and Technology, Northeast Agricultural University, Harbin, China

**Keywords:** LEF1, miR-17-92 cluster host gene (MIR17HG), Wnt/β-catenin pathway, target gene, cell proliferation

## Abstract

The miR-17-92 cluster is involved in animal development and homeostasis, and its dysregulation leads to human diseases such as cancer. In the present study, we investigated the functional link between miR-17-92 cluster and Wnt/β-catenin signaling pathway in ICP2 and DF1 cells. We demonstrated that ectopic expression of either LEF1 or β-catenin increased the promoter activity of the miR-17-92 cluster host gene (MIR17HG) and combined ectopic expression of LEF1 and β-catenin further enhanced the promoter activity; while knockdown of either LEF1 or β-catenin reduced the MIR17HG promoter activity. Both LEF1 and β-catenin could directly bind to the MIR17HG promoter. Furthermore, we demonstrated that low doses of lithium chloride (LiCl), an activator of Wnt/β-catenin signaling pathway, increased MIR17HG promoter activity and the endogenous expression of the miR-17-92 cluster, while high doses of LiCl had the opposite effects. Treatment with XAV-939, an inactivator of the Wnt/β-catenin pathway, reduced the endogenous expression of miR-17-92 cluster. Finally, we found that low doses of LiCl promoted the proliferation of ICP2 and DF1 cells, while high doses of LiCl inhibited the proliferation of ICP2 and DF1 cells. Taken together, our results reveal that MIR17HG is a target of LEF1 and the Wnt/β-catenin pathway and suggest that the miR-17-92 cluster may, at least in part, mediate the proliferation-promoting effect of the Wnt/β-catenin pathway in cell proliferation.

## Introduction

MicroRNAs (miRNAs) are single-stranded RNA molecules of 19–22 nucleotides in length, which regulate gene expression post-transcriptionally (Bartel, 2004; Eulalio et al., 2008). They typically bind to the 3′-untranslated region (3′UTR) of target mRNAs, resulting in translational repression or mRNA degradation (Bartel, 2009). To date, accumulating evidence has demonstrated that miRNAs exert crucial roles in cell proliferation, differentiation, apoptosis, development, and oncogenesis.

MicroRNAs genes are scattered across chromosomes either individually or in clusters, in which two or more miRNAs are transcribed from adjacent miRNA genes within a short distance. The polycistronic miRNA cluster, miR-17-92, is one of the best-studied miRNA clusters. The human miR-17-92 cluster is located in an intron of the miR-17-92 cluster host gene (MIR17HG) on chromosome 13 (13q31.3). The primary transcript of miR-17-92 (pri-miR-17-92) is an approximately 0.8-kb-long polycistron and is sequentially processed to produce seven different mature miR-17-92 cluster members (miR-17-3p, miR-17-5p, miR-18, miR-19a, miR-19b, miR-20, and miR-92) (He et al., 2005). Previous studies have shown that the miR-17-92 cluster is involved in multiple biological processes, including cell proliferation, differentiation, apoptosis, development, and oncogenesis (Ventura et al., 2008; Mu et al., 2009; Olive et al., 2009). The miR-17-92 cluster is frequently amplified in various types of lymphomas, including B-cell lymphoma, follicular lymphoma, and some solid tumors (Ota et al., 2004; He et al., 2005).

The transcriptional regulation of the miR-17-92 cluster has been extensively studied over the past decade. The c-Myc oncogene was the first transcription factor shown to directly bind and activate the transcription of MIR17HG (O’donnell et al., 2005; Kumar et al., 2013). To date, a number of other transcription factors have been identified as direct regulators of MIR17HG transcription. Among the identified regulators, c-Myc, N-Myc, STAT3, STAT5, MYCN, MYB, Spi-1, Fli-1, Pim-1, cyclin D1, AML1, ETS1, ETS2, NKX3.1, and E2F family members activate (Fontana et al., 2007; Sylvestre et al., 2007; Yu et al., 2008; De Brouwer et al., 2012; Feuermann et al., 2012; Kayali et al., 2012; Thomas et al., 2013; Kabbout et al., 2014; Yang et al., 2015; Spagnuolo et al., 2019), whereas p53 and C/EBPβ suppress the transcription of MIR17HG (Yan et al., 2009; Yan et al., 2016).

The Wnt/β-catenin signaling pathway is essential for regulating cell proliferation and differentiation (Teo and Kahn, 2010; Kahn, 2014). β-catenin is a key mediator of canonical Wnt signaling and acts as a transcriptional co-activator. Upon activation of the Wnt/β-catenin pathway, β-catenin enters the nucleus and interacts with transcription factors T-cell and lymphoid enhancer factors (TCF/LEF1) to activate and/or repress transcription of specific target genes (Clevers, 2006).

Our group previously closed the genomic gap upstream of the chicken miR-17-92 cluster using genome walking (Cheng et al., 2016). Our bioinformatics analysis identified a conserved potential LEF1 binding site located 74 bp upstream of the predicted transcriptional start site of MIR17HG promoter. This led us to hypothesize that the miR-17-92 cluster is a target of the Wnt/β-catenin/LEF1 signaling pathway. In the present study, we investigated whether the Wnt/β-catenin/LEF1 pathway regulates the transcription of MIR17HG. We demonstrated that LEF1 and β-catenin bind to and regulate MIR17HG promoter activity, and that activation of the Wnt/β-catenin pathway increases the MIR17HG promoter activity as well as the endogenous expression of miR-17-92 cluster, whereas inactivation of the Wnt/β-catenin pathway reduces miR-17-92 cluster expression.

## Materials and Methods

### Cell Cultures and Treatments

The chicken DF1 cell line was purchased from the Institute of Biochemistry and Cell Biology, Chinese Academy of Sciences. DF1 cells were cultured in Dulbecco’s Modified Eagle’s Medium (DMEM) with high glucose formulation (Gibco, United States) supplemented with 10% fetal bovine serum (BI, Germany), 100 units/ml penicillin, and 100 μg/ml streptomycin, and incubated at 37°C, 5% CO_2_. Immortalized chicken preadipocytes (ICP2) were generated in our lab (Wang et al., 2017). ICP2 cells were maintained in DMEM:nutrient mixture F12 (DMEM/F12) (Gibco, United States) supplemented with 10% fetal bovine serum (BI, Germany), 100 units/ml penicillin, and 100 μg/ml streptomycin, and incubated at 37°C, 5% CO_2_.

To activate the Wnt/β-catenin signaling pathway, ICP2 and DF1 cells were treated with lithium chloride (LiCl) (Sigma-Aldrich, St. Louis, MO, United States) for 24 h in DMEM and DMEM/F12 at concentrations of 3, 15, and 30 mM; sodium chloride (NaCl) was used as a negative control. To inactivate the Wnt/β-catenin signaling pathway, ICP2 and DF1 cells were treated with 10 μM XAV-939 (MedChem Express, NJ, United States) dissolved in DMSO (Sigma-Aldrich, St. Louis, MO, United States) for 24 h; DMSO was used as a negative control.

### RNA Extraction and qRT-PCR Assays

Total RNA was isolated from ICP2 and DF1 cells using Trizol reagent (Invitrogen, Carlsbad, CA, United States). For miRNA expression analysis, the extracted RNA was reverse transcribed using the PrimeScript^TM^ RT reagent Kit with gDNA Eraser (Perfect Real Time) (Takara, Japan) with miRNA-specific stem-loop RT primers (Table 1) according to the manufacturer’s protocol. The relative expression of miRNAs was determined by real-time PCR using FastStart Universal SYBR Green Master [Rox] (Roche, United States) with miRNA-specific forward primers and a universal primer (URP). U6 snRNA was used for normalization and the 2^−ΔΔCt^ method was used to analyze the relative miRNA expression levels. For mRNA expression analysis, total RNA (1 μg) was reverse-transcribed into cDNA using the PrimeScript^TM^ RT reagent Kit with gDNA Eraser (Takara, Japan), and the relative mRNA expression was determined as described above. TBP was used for normalization. All primers for stem-loop qRT-PCR and qRT-PCR analyses are shown in Table 1.

**Table 1:** Primers used for reverse transcriptase PCR and quantitative real-time PCR.

Type	Primer name	Primer sequence
miRNA qRT-PCR primer	miRNA-17-5p forward	ACACTCCAGCTGGGCAAAGTGC TTACAGTGCA
	miRNA-17-5p RT	CTCAACTGGTGTCGTGGAGTCG GCAATTCAGTTGAGACTACCTG
	miRNA-17-3p forward	ACACTCCAGCTGGGACTGCAGT GAAGGC
	miRNA-17-3p RT	CTCAACTGGTGTCGTGGAGTCG GCAATTCAGTTGAGACAAGTGC
	U6-RT	AACGCTTCACGAATTTGCGT
	U6 forward	CTCGCTTCGGCAGCACA
	U6 reverse	AACGCTTCACGAATTTGCGT
	URP	TGGTGTCGTGGAGTCG
mRNA qRT-PCR	pri-miRNA-17-92-F	CATCTACTGCCCTAAGTGCT CCTT
primer	pri-miRNA-17-92-R	GCTTGGCTTGAATTATTGGATGA
	PCNA-F	GTGCTGGGACCTGGGTT
	PCNA-R	CGTATCCGCATTGTCTTCTG
	CyclinD1-F	CTCGGAGCTACCTGCATGTTT
	CyclinD1-R	GTTTACGGATGATCTGTTTGGTG
	TBP-F	GCGTTTTGCTGCTGTTATTATGAG
	TBP-R	TCCTTGCTGCCAGTCTGGAC
	LEF1-F	GTACAGCCTTCTCACGCAGT
	LEF1-R	GAAAACCAGCCAAGAGGTGG
	β-catenin-F	CGCCATTTTAAGCCTCTCGC
	β-catenin-R	CCTTTCAGAGACTGTGGCACG
CHIP qRT-PCR	CHIP promoter-F	CGCTCGCTCGCTCGGTGCAT
primer	CHIP promoter-R	AGCCCCGCTCCGCCCTCATT

### Plasmid Construction

To construct a luciferase reporter with the MIR17HG promoter, a 714-bp chicken genomic DNA fragment (–217/+496 relative to the predicted transcriptional start site of MIR17HG) was synthesized by GENEWIZ (Suzhou, China) and cloned into the luciferase reporter vector, pGL3-Basic (Promega, Madison, WI), to yield the MIR17HG promoter reporter (pGL3-WT-MIR17HG-Luc). The potential TCF/LEF1 binding site of the pGL3-WT-MIR17HG-Luc was mutated from TTTGTT to GAATTC using DNA synthesis, and the resulting reporter construct was designated as pGL3-MT-MIR17HG-Luc. For expression vector constructs, the full-length CDS of LEF1 (NM_205013.2) was amplified from the pooled chicken cDNA using PCR with the forward primer: 5′-(G/A)NNATGNCGCAGCTGCCGGGGGCCGGGG-3′ and the reverse primer: 5′-GATGTAGGCAGCTGTCATTCTGGGG-3′ and cloned into the pEASY-Blunt-M2 vector to generate the LEF1 expression vector (pEASY-Blunt-M2-LEF1). The full-length CDS of β-catenin (NM_205081.1) was amplified using PCR with the forward primer: 5′-CGGAATTCGGGCTGACTTGATGGAGTTGGA-3′ and the reverse primer: 5′-GGGGTACCCAACTGATTACTGTCACCTGG-3′ and cloned into pCMV-HA vector in the EcoR I/Kpn I sites to yield pCMV-HA-WT-β-catenin. In addition, to generate the expression vector for the constitutively active mutant of β-catenin (Δ45-β-catenin), the point mutation was generated from pCMV-HA-WT-β-catenin by using Site-directed Gene Mutagenesis Kit (Beyotime) with the primers 5′-ACGACAACTGCTCCCTTGAGTGGCAAAGGAA-3′ and 5′-TTCCTTTGCCACTCAAGGGAGCAGTTGTCGT-3′, and the Δ45-β-catenin expression vector was designated as pCMV-HA-Mut-β-catenin. All constructs were confirmed by DNA sequencing and successful expression was confirmed by western blotting (Supplementary Figure S1).

### Small Interfering RNA (siRNA)

LEF1 siRNA (si-LEF1), β-catenin siRNA (si-β-catenin) and control siRNA (si-NC) were designed and synthesized by GenePharma (Suzhou, China). The following siRNA sequences were used: si-NC sense (5′–3′) UUCUCCGAACGUGUCACGUTT, si-NC antisense (5′–3′) ACGUGACACGUUCGGAGAATT,si-LEF1 sense (5′–3′) GCUAUCAACCAGAUUCUUGTT, and si-LEF1 antisense (5′–3′) CAAGAAUCUGGUUGAUAGCTT, si-β-catenin sense (5′–3′) GCUUUAGGACUCCACCUUATT,si-β-catenin antisense (5′–3′) UAAGGUGGAGUCCUAAAGCTT. DF1 or ICP2 cells were plated at 1 × 10^5^ cells per well in 12-well plates. After 24 h of culture, the cells were transfected with si-LEF1, si-β-catenin, and si-NC, respectively. At 48 h post-transfection, total RNA and protein were extracted from these transfected cells. The knockdown efficiency of LEF1 and β-catenin was tested by qRT-PCR and Western blot analysis.

### Luciferase Reporter Gene Assay

The luciferase reporter plasmids (Topflash and Fopflash) were obtained from the Addgene repository. The Topflash harbors three optimal TCF/LEF1-binding sites, and Fopflash harbors three mutated TCF/LEF1-binding sites. To detect Wnt/β-catenin signaling pathway activity, ICP2 and DF1 cells were plated at 2 × 10^4^ cells per well in 48-well plates. After 24 h of culture, the cells were transfected with either 200 ng of Topflash or Fopflash, along with 4 ng of pRL-TK. For MIR17HG promoter activity analysis, ICP2 cells were plated at 5 × 10^4^ cells per well in 24-well plates. After 24 h of culture, the cells were transfected with 500 ng pGL3-WT-MIR17HG-Luc or pGL3-MT-MIR17HG-Luc and 10 ng pRL-TK. For transcriptional regulation analysis, cells were co-transfected with 200 ng pGL3-WT-MIR17HG-Luc or pGL3-MT-MIR17HG-Luc and 200 ng of pEASY-Blunt-M2-LEF1, pCMV-HA-WT-β-catenin, pCMV-HA-Mut-β-catenin, pEASY-Blunt-M2/pCMV-HA, 10 pmol of si-LEF1, si-β-catenin, or si-NC. All cells were transfected using Lipofectamine 2000 (Invitrogen, United States) following the manufacturer’s protocol. At 48 h after transfection, the cells were harvested and luciferase activity was measured using the dual-luciferase reporter assay system (Promega, United States). The luciferase activities were normalized to the corresponding Renilla luciferase activity values.

### Immunofluorescence

Immunofluorescence was performed using Immunofluorescence Application Solutions Kit (Cell Signaling Technology, United States) according to the manufacturer’s instructions. In brief, Cells cultured on coverslips were treated at the indicated concentrations of LiCl and fixed with 4% (w/v) paraformaldehyde for 15 min. The cells were then washed with PBS and blocked in blocking buffer for 60 min at room temperature. Then cells were incubated with anti-β-catenin antibody (CST, 8480S) or anti-LEF1 antibody (Abcam, ab137872) overnight at 4°C, respectively. The cells were stained with FITC-conjugated anti-rabbit IgG (Transgen biotech, China) for 60 min at room temperature, followed by nuclei counter-staining with Hoechst 33342 for 5 min at room temperature (Beyotime, China). Fluorescent images were examined under a confocal laser microscope (Leica Microsystems, Bannockburn, IL, United States).

### Western Blot Analysis

Nuclear proteins were extracted from ICP2 and DF1 cells treated with LiCl, using the NE-PER nuclear extraction kit (Pierce, United States). Cells were lysed in RIPA buffer, supplemented with PMSF (RIPA:PMSF = 100:1). Cell lysates were separated by 10% SDS-PAGE and transferred onto nitrocellulose membrane (Millipore, United States). The blots were incubated with the following primary antibodies: anti-LEF1 (1:1000; Abcam, United Kingdom), anti-β-catenin (1:1000, CST, United States), anti-HA (1:500; Beyotime, China), anti-Myc (1:500; Beyotime, China). β-actin (1:4000; proteintech, China) and Lamin B_1_ (1:3000; proteintech, China) were used as a loading control. Immunoreactive bands were detected using the ECL plus detection kit (HaiGene, China).

### Electrophoretic Mobility Shift Assay (EMSA)

Nuclear proteins were extracted from DF1 cells transfected with pEASY-Blunt-M2-LEF1 using the NE-PER nuclear extraction kit (Pierce, United States). Binding activity of LEF1 to the probes was determined using the LightShift Chemiluminescent EMSA Kit (Pierce, United States). Biotin-labeled probes harboring the potential TCF/LEF1 binding site and the corresponding mutant probes were synthesized by Genewiz. The sequences of forward and reverse single-stranded probes are shown in Table 2. Forward and reverse single-stranded probes were annealed to form double-stranded DNA probes. The labeled double-stranded probes were incubated with 3 μl NE-PER nuclear extracts for 30 min at room temperature. For binding competition experiments, 50 or 100-fold molar excess of unlabeled double-stranded wild type or mutant probes were added into the binding reactions immediately prior to addition of the labeled probes. For supershift assay, 1 μg anti-Myc (CST, United States) or normal mouse IgG (Beyotime, China) was added to the binding reactions. Normal mouse IgG was used as a negative control for non-specific binding. The DNA–protein complexes were resolved on a 6% native polyacrylamide gel and detected using ECL development.

**Table 2:** List of probes used in EMSA analysis.

Probe name	Sequences 5′–3′
WT-F	TCGGAAGCACTTTGTTTTCTATTCT
WT-R	AGAATAGAAAACAAAGTGCTTCCGA
MT-F	TCGGAAGCAC***gaat***T***c***TTCTATTCT
MT-R	AGAATAGAA***g***A***attcg***TGCTTCCGA

*LEF1 binding sites are underlined; substitution mutations are presented in lower case italic letters.*

### Chromatin Immunoprecipitation Assay

Chromatin immunoprecipitation (ChIP) assay was performed using the ChIP assay kit (Cell Signaling Technology, United States) according to the manufacturer’s instructions. ICP2 cells were transfected with pEASY-Blunt-M2-LEF1, pCMV-HA-WT-β-catenin, or pEASY-Blunt-M2/pCMV-HA. At 48 h after transfection, cells were fixed with 1% formaldehyde for 10 min at room temperature and quenched with 125 mM glycine for 5 min at room temperature. The DNA was digested with 0.5 μl micrococcal nuclease into an average fragment size of 100–900 bp. The sample was incubated with anti-β-catenin (CST, United States), anti-LEF1 (Abcam, United Kingdom), or the normal rabbit IgG (CST, United States) as a control. The immunoprecipitates were collected, washed, eluted, and then the cross-links were reversed. The DNA was purified and analyzed by qPCR using the site-specific PCR primers (Table 1). Non-immunoprecipitated DNA (2%) was used as input control. ChIP qPCR data were normalized to input chromatin DNA and then presented as fold enrichment over the negative control using the ΔΔCt equation (Tatler et al., 2016).

### Cell Proliferation Assay

Cell proliferation was determined using the Cell Counting Kit-8 (CCK-8) (DOJINDO, Japan) according to the manufacturer’s instructions. Briefly, ICP2 and DF1 cells were seeded onto 48-well plates at 1 × 10^4^ cells/well. After 12 h, the cells were treated with different LiCl concentrations (3, 15, and 30 mM) for 0, 24, 48, 72, and 96 h. The control cells were treated with NaCl (3, 15, and 30 mM). At designated time points, 20 μL of CCK-8 solution was added to each well of the plates, followed by 1.5 h incubation at 37°C. The absorbance was measured at 450 nm. All experiments were repeated at least three times.

### Data Analysis

The data are shown as the mean ± SD. Comparisons between two groups were performed using two-tailed Student *t*-test using GraphPad Prism Software. Statistical differences were considered significant when *P* < 0.05.

## Results

### LEF1 Regulates MIR17HG Promoter Activity

**FIGURE 1:**
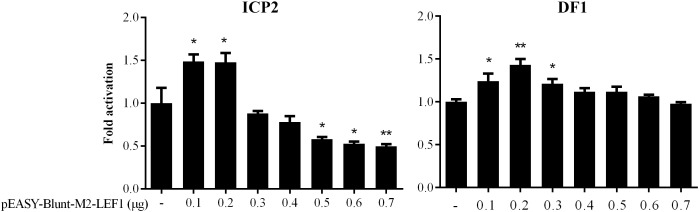
MIR17HG promoter activity is regulated by LEF1. Luciferase reporter assays showing the effect of ectopic expression of LEF1 on MIR17HG promoter activity in ICP2 and DF1 cells. Cells were transfected with the indicated amounts of LEF1 expression plasmid (pEASY-Blunt-M2-LEF1). The total amount of DNA in each transfection was kept constant by adding empty vector DNA (pEASY-Blunt-M2). Two days after transfection, Firefly luciferase activity was measured and normalized to Renilla luciferase activity. The data are presented as the mean ± SD of triplicate experiments. ^∗^*P* < 0.05 and ^∗∗^*P* < 0.01.

Our previous bioinformatics analysis showed that a potential LEF1 binding site within the MIR17HG promoter region was conserved among chicken, mouse, and human species (Cheng et al., 2016), suggesting that MIR17HG may be a target gene of LEF1. To test whether LEF1 regulates MIR17HG, a 714-bp promoter fragment containing the potential LEF1 binding site was amplified and cloned upstream of the firefly luciferase reporter, pGL3-basic vector. The resulting vector was designated pGL3-WT-MIR17HG-Luc. We transfected pGL3-WT-MIR17HG-Luc with or without the LEF1 expression plasmid (pEASY-Blunt-M2-LEF1) into ICP2 and DF1 cells and then measured the luciferase activity. The results showed that compared to the empty vector pEASY-Blunt-M2, at low doses (0.1–0.2 μg), pEASY-Blunt-M2-LEF1 increased the MIR17HG promoter activity by over 1.4-fold in ICP2 and DF1 cells (*P* < 0.05), whereas at high doses (0.5–0.7 μg), pEASY-Blunt-M2-LEF1 decreased the promoter activity in ICP2 cells (*P* < 0.05) (Figure 1). These reporter assay results suggest that ectopic expression of LEF1 regulates MIR17HG promoter activity. To determine whether the potential LEF1 binding site is required for the regulation of MIR17HG transcription by LEF1, the consensus LEF1 binding site CTTTGTT was substituted by CGAATTC in the MIR17HG promoter reporter construct (pGL3-WT-MIR17HG-Luc), and the resulting LEF1 binding site-mutated reporter construct was designated pGL3-MT-MIR17HG-Luc. DF1 or ICP2 cells were transfected with pGL3-MT-MIR17HG-Luc with or without the LEF1 expression plasmid, and the luciferase activity was measured. The reporter assay showed that ectopic expression of LEF1 had no activating effect on the promoter activity of pGL3-MT-MIR17HG-Luc (*P* > 0.05) (data not shown). Collectively, these results suggest that this LEF1 binding site is involved in the regulation of MIR17HG.

### LEF1 Directly Binds to the MIR17HG Promoter

**FIGURE 2:**
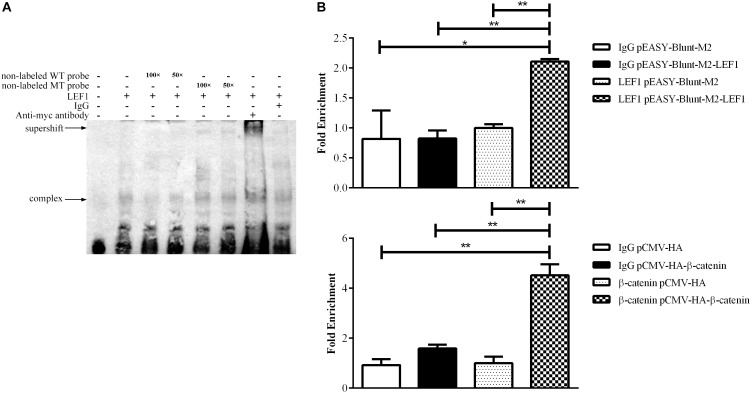
LEF1 directly binds to the MIR17HG promoter. **(A)** Electrophoretic mobility shift (EMSA) and supershift assays showing binding of β-catenin/LEF1 to the MIR17HG promoter. **(B)** Chromatin immunoprecipitation (ChIP) assays showing binding of LEF1 and β-catenin to the MIR17HG promoter using antibodies against LEF1 and β-catenin, respectively, in ICP2 cells. ^∗^*P* < 0.05 and ^∗∗^*P* < 0.01.

To validate whether LEF1 directly binds to the potential LEF1 binding site within the MIR17HG promoter region, we performed EMSA of nuclear protein extracts from the LEF1-transfected DF1 cells with biotin-labeled wild-type (WT-LEF1) oligonucleotide probes corresponding to the MIR17HG promoter (–85/–61, relative to the predicted transcriptional start site of MIR17HG) (Table 2). A specific protein–DNA complex band was detected when the wild-type probe was incubated with the nuclear protein extracts. Addition of 50-fold or 100-fold excess of the unlabeled wild-type probe, but not the unlabeled mutant probe, completely eliminated protein binding to the labeled wild-type probe. Additionally, the protein–DNA complex was supershifted by the LEF1 antibody but not normal IgG (Figure 2A). Furthermore, we performed a ChIP assay to confirm *in vivo* binding of β-catenin/LEF1 to this LEF1 binding site in ICP2 cells. ICP2 cells were transfected with pEASY-Blunt-M2-LEF1, pCMV-HA-WT-β-catenin, or pEASY-Blunt-M2/pCMV-HA. ChIP assay was performed using anti-β-catenin and anti-LEF1 antibodies as well as normal rabbit IgG control. Enriched DNA was analyzed using quantitative PCR with a specific pair of CHIP qRT-PCR primers (Table 1). As demonstrated in Figure 2B, the MIR17HG promoter fragment was significantly enriched by over 2.5- and 3.8-fold in the DNA immunoprecipitated by the anti-LEF1 and anti-β-catenin antibodies, respectively, compared with the normal rabbit IgG control (*P <* 0.01). As expected, the MIR17HG promoter fragment was not enriched in any one of the negative controls (pEASY-Blunt-M2 and pCMV-HA) (*P* > 0.05). Taken together, all these data suggest that LEF1 and β-catenin specifically bind to and regulate the MIR17HG promoter.

### The Wnt/β-Catenin Pathway Regulates MIR17HG Promoter Activity

**FIGURE 3:**
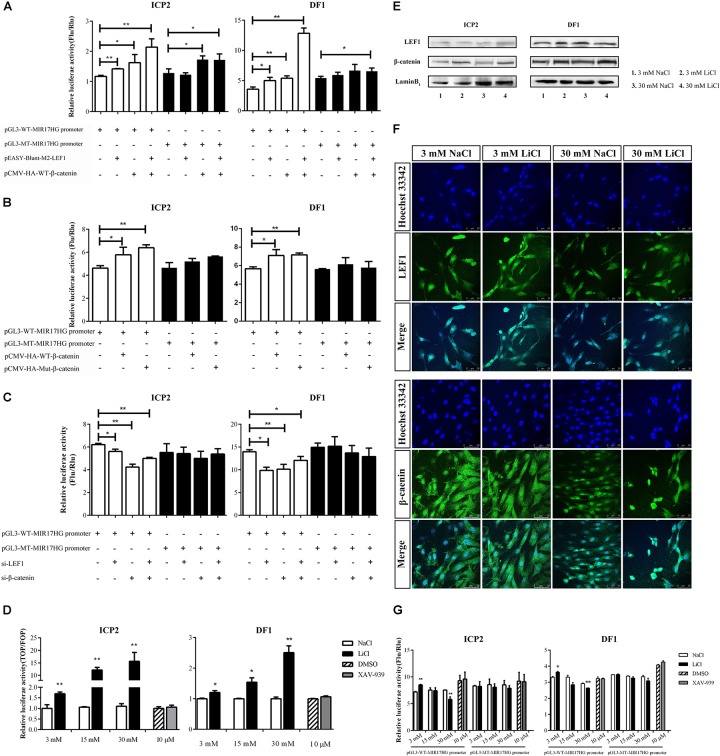
MIR17HG is activated by the Wnt/β-catenin pathway. **(A)** Luciferase reporter assays showing the effects of ectopic expression of LEF1 and β-catenin on MIR17HG promoter activity. **(B)** Luciferase reporter assays showing the effect of ectopic expression of β-catenin on MIR17HG promoter activity. **(C)** Luciferase reporter assays showing the effects of knockdown of either LEF1 or β-catenin on MIR17HG promoter activity. **(D)** Luciferase reporter assay demonstrating that the Top/Fopflash ratio was affected by various concentrations of LiCl and XAV-939 for 24 h in ICP2 and DF1 cells. The Firefly luciferase activity values were normalized to a Renilla transfection control. Three independent assays were performed. ^∗^*P* < 0.05 and ^∗∗^*P* < 0.01. Western blot analysis **(E)** and Immunofluorescence **(F)** showing nuclear levels of β-catenin and LEF1 in ICP2 and DF1 cells treated at indicated concentrations of LiCl. **(G)** Luciferase reporter assays showing the effects of LiCl treatment at the indicated concentrations (3, 15 and 30 mM) and XAV-939 (10 μM) on the MIR17HG promoter activity. The Firefly luciferase activity was normalized to Renilla luciferase activity. The data are presented as the mean ± SD of triplicate experiments. *^∗^P* < 0.05 and *^∗∗^P* < 0.01.

LEF1 is a key mediator of the Wnt/β-catenin signaling pathway, which interacts with β-catenin to regulate Wnt target gene expression. To validate whether LEF1 and β-catenin interact to regulate MIR17HG, we determined the effect of LEF1 and β-catenin interaction on MIR17HG promoter activity. ICP2 and DF1 cells were transfected with pGL3-WT-MIR17HG-Luc along with LEF1 and β-catenin expression vectors either alone or in combination, and the luciferase activity was determined. The results showed that ectopic expression of LEF1 and β-catenin enhanced the promoter luciferase activity of pGL3-WT-MIR17HG-Luc (*P* < 0.05), and combined ectopic expression of LEF1 and β-catenin further enhanced the promoter activity of pGL3-WT-MIR17HG-Luc, compared with LEF1 and β-catenin alone (Figure 3A), suggesting LEF1 and β-catenin interact to regulate the promoter activity. As expected, both β-catenin and Δ45-β-catenin increased the MIR17HG promoter activity (Figure 3B) but had no activating effect on the promoter activity of the LEF1 binding site-mutated reporter pGL3-MT-MIR17HG-Luc (*P* > 0.05). To validate our findings, we also performed LEF1 and β-catenin knockdown experiments. We first confirmed the knockdown efficiency of LEF1 and β-catenin at both mRNA and protein levels using qRT-PCR and Western blotting, respectively (Supplementary Figure S2). Then, we cotransfected pGL3-WT-MIR17HG-Luc and si-LEF1 or si-β-catenin into ICP2 and DF1 cells and determined the luciferase activity. The results showed that knockdown of either LEF1 or β-catenin by siRNA in both ICP2 and DF1 cells decreased the MIR17HG promoter activity (*P* < 0.05) (Figure 3C). Taken together, these data indicate that that LEF1 and β-catenin interact to regulate the promoter activity of MIR17HG.

To further validated the finding that the Wnt/β-catenin pathway regulates MIR17HG, we examined the effects of Lithium chloride (LiCl), an activator of Wnt/β-catenin signaling (Klein and Melton, 1996), and XAV-939, an inactivator of Wnt/β-catenin signaling (Huang et al., 2009), on the promoter activity of the MIR17HG in ICP2 and DF1 cells using the reporter gene assay. Ratios between the luciferase activities of Topflash and Fopflash reporters (Top/Fop ratio) are widely used to measure Wnt/β-catenin signaling activity (Korinek et al., 1997). ICP2 and DF1 cells were transfected with indicated reporter plasmids and treated with LiCl at concentrations of 3, 15, and 30 mM for 24 h, and the control cells were treated with NaCl at concentrations of 3, 15, and 30 mM. Reporter gene assay showed LiCl treatment increased the Top/Fop ratio in a dose-dependent manner in ICP2 and DF1 cells (*P* < 0.01), indicating that LiCl treatment can activate Wnt/β-catenin signaling pathway. Unlike LiCl treatment, XAV-939 treatment exhibited no significant effect on Top/Fop ratio (*P* > 0.05) (Figure 3D). Consistent with the reporter gene assay results, Western blotting and immunofluorescence showed that nuclear β-catenin was stabilized by LiCl (3 mM and 30 mM) and LEF1 protein level was elevated by LiCl (30 mM) (Figure 3E,F and Supplementary Figure S3). As shown in Figure 3G, LiCl treatment at a low concentration (3 mM) resulted in a 1.2-fold increase in the luciferase activity of the MIR17HG promoter (*P* < 0.05), whereas LiCl treatment at a high concentration (30 mM) led to a decrease in the luciferase activity of the MIR17HG promoter (*P* < 0.01) (Figure 3G). In contrast, XAV-939 treatment had no significant effect on the promoter activity of the MIR17HG (*P* > 0.05) (Figure 3G). As expected, LiCl treatment at the three different concentrations had no significant effect on the luciferase activity of the LEF1 binding site-mutated MIR17HG promoter (*P* > 0.05) (Figure 3G). Collectively, these data suggest that the Wnt/β-catenin/LEF1 signaling pathway regulates MIR17HG.

### The Wnt/β-Catenin Pathway Regulates the Endogenous Expression of the miR-17-92 Cluster

**FIGURE 4:**
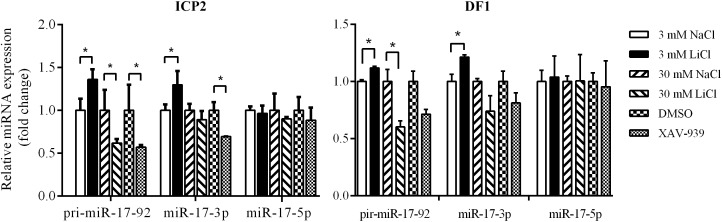
Wnt/β-catenin signaling regulates the endogenous expression of the miR-17-92 cluster. Quantitative RT–PCR analysis showing relative expression of pri-miRNA-17-92, miR-17-3p, and miR-17-5p in ICP2 and DF1 cells treated with LiCl (3 and 30 mM, respectively) and XAV-939 for 24 h. The plotted bar graph values were normalized to the NaCl and DMSO treatment control, respectively. The relative expression levels of mature miRNA and pri-miRNA-17-92 were normalized to U6 small nuclear RNA (snRNA) and TBP, respectively. Mean ± SD, ^∗^*P* < 0.05.

To validate the above results, we used qRT-PCR to assess the expression of the primary miR-17-92 transcript (pri-miR-17-92) and two mature miR-17-92 cluster members (miR-17-3p and miR-17-5p) in the ICP2 and DF1 cells whose Wnt/β-catenin pathway is activated or inactivated by LiCl treatment or XAV-939 treatment. Real-time RT-PCR analysis showed that 3 mM LiCl treatment increased the expression of pri-miR-17-92 and miR-17-3p by 1.36-fold and 1.30-fold compared with 3 mM NaCl-treated cells, respectively (*P* < 0.05) (Figure 4). However, 30 mM LiCl treatment decreased the expression of pri-miR-17-92 in ICP2 and DF1 cells (*P* < 0.05) (Figure 4). In contrast, XAV-939 treatment reduced the expression levels of pri-miR-17-92 and miR-17-3p in ICP2 cells (*P* < 0.05) (Figure 4). Taken together, these data suggest that Wnt/β-catenin signaling regulates the endogenous expression of the miR-17-92 cluster in ICP2 and DF1 cells.

### The Wnt/β-Catenin Pathway Regulates ICP2 and DF1 Cell Proliferation

**FIGURE 5:**
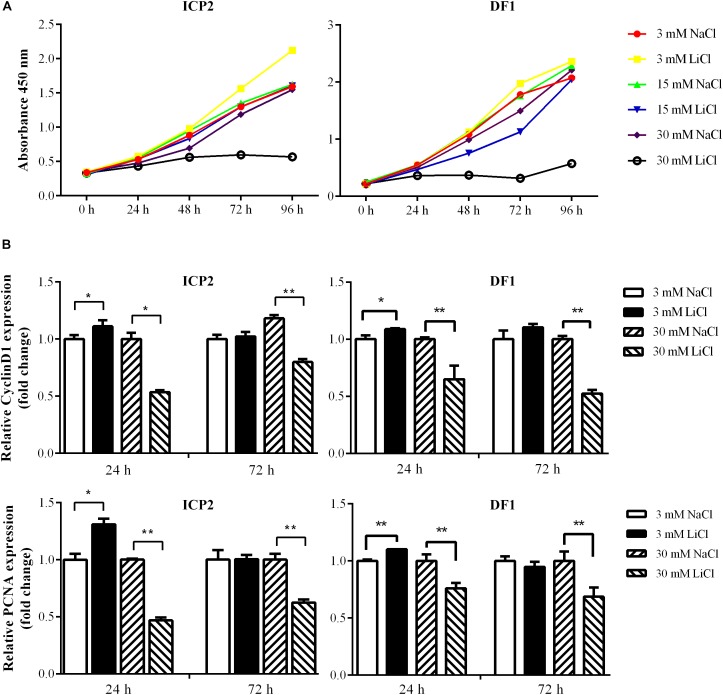
Effects of activation and inactivation of the Wnt/β-catenin signaling pathway on ICP2 and DF1 cells proliferation. **(A)** Effects of various concentrations of LiCl on the proliferation of ICP2 and DF1 cells. Cells were treated at indicated concentrations of LiCl and cell proliferation was assessed at 0, 24, 48, 72 and 96 h using the CCK-8 kit. **(B)** Effects of LiCl treatment at low (3 mM) and high (30 mM) concentrations on the expression of proliferation marker genes in ICP2 and DF1 cells. Cells were treated at various concentrations of LiCl, total RNA was isolated, and the gene expression of Cyclin D1 and PCNA were assessed at the indicated time points using qRT-PCR. The relative gene expression levels were normalized to TBP. Fold change is relative to the control group (NaCl treated) at 24 h. All data are representative of three independent experiments and shown as the mean ± SD. ^∗^*P* < 0.05; ^∗∗^*P* < 0.01; determined by two-tailed Student’s *t*-test.

The Wnt/β-catenin signaling pathway is essential for regulating cell proliferation (Teo and Kahn, 2010) and our previous study demonstrated that overexpression of the miR-17-92 cluster promoted cell proliferation (Zhang et al., 2017). In the present study, we demonstrated that the Wnt/β-catenin signaling pathway upregulated miR-17-92 cluster expression (Figure 4). To confirm the functional link between the Wnt/β-catenin pathway and miR-17-92 cluster, we tested whether activation of the Wnt/β-catenin signaling pathway promotes cell proliferation. ICP2 and DF1 cells were treated with LiCl, and cell proliferation was measured using the Cell Counting Kit 8 (CCK-8). The results showed that 3 mM LiCl treatment significantly increased, and 30 mM LiCl treatment significantly decreased the proliferation of ICP2 and DF1 cells at 48, 72, and 96 h (Figure 5A). In parallel, we examined the expression of the proliferation markers cyclin D1 and PCNA at 24 and 72 h. The expression analysis demonstrated that 3 mM LiCl treatment significantly increased the expression of cyclin D1 and PCNA at 24 h (*P* < 0.05), but not at 72 h. Conversely, 30 mM LiCl treatment significantly decreased the expression of cyclin D1 and PCNA at 24 and 72 h (*P* < 0.01) (Figure 5B). Considering that 3 mM LiCl treatment increased miR-17-92 cluster expression, but 30 mM LiCl treatment decreased miR-17-92 cluster expression (Figure 4). We conclude that Wnt/β-catenin signaling pathway promotes cell proliferation at least in part via upregulation of the miR-17-92 cluster.

## Discussion

Several signaling pathways have been shown to regulate miR-17-92 cluster expression, including Notch and Shh. The Shh signaling pathway can upregulate miR17-92 cluster expression through activation of c-Myc (Liu et al., 2013) and N-Myc (Northcott et al., 2009). The MIR17HG promoter contains a potential binding site for the Notch signaling effector, HES1, and previous studies have shown that Notch-1 overexpression upregulates miR-19 expression while Notch-1 knockdown reduces miR-19 expression in normal thyroid cells (Fuziwara and Kimura, 2014). Furthermore, the MIR17HG promoter also contains a conserved Smad-binding element, and both reporter gene and *in vivo* ChIP assays showed that BMP and TGF-β signaling pathways directly increases the transcription of miR-17-92 via activation of R-Smad (Wang et al., 2010; Sun et al., 2013; Luo et al., 2014). In the present study, we identified MIR17HG as a target of LEF1 and Wnt/β-catenin pathway (Figure 1–4). Our results showed that LEF1 and β-catenin could bind to and regulate the MIR17HG promoter (Figure 2). Consistent with our result, a previous study showed that β-catenin and TCF4, a member of T-cell and lymphoid enhancer (TCF/LEF) factor family, could bind to and regulate MIR17HG promoter in colorectal cancer (Li et al., 2016). These data suggest that the miR-17-92 cluster is an evolutionarily conserved target of the Wnt signaling pathway.

**FIGURE 6:**
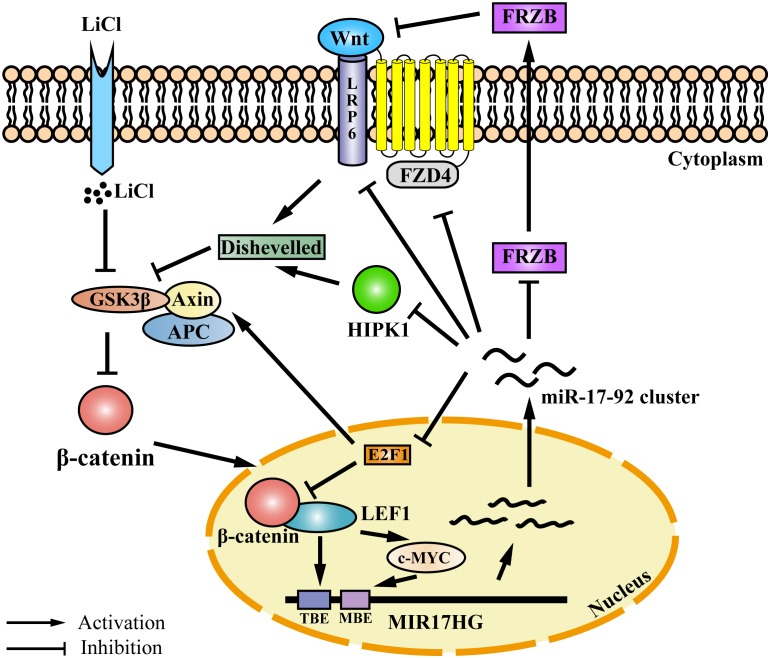
Proposed model for an auto-regulatory feedback loop between the miR-17-92 cluster and the Wnt/β-catenin/LEF1 signaling pathway. The Wnt/β-catenin/LEF1 pathway activates the miR-17-92 cluster expression by two mechanisms. In one mechanism, LEF1 directly binds to the LEF1 binding element (TBE) in the MIR17HG promoter and activates the transcription of MIR17HG. In the other mechanism, the Wnt downstream targets, including c-MYC and cyclin D1, bind to the MIR17HG promoter and activate the transcription of MIR17HG. The miR-17-92 cluster, in turn, modulates Wnt/β-catenin**/**LEF1 signaling activity by several mechanisms. The miR-17-92 cluster can target agonists (FRZB and RP6) and antagonists (FZD6 and HIPK1) of the Wnt/β-catenin signaling pathway. Moreover, the miR-17-92 cluster can modulate Wnt/β-catenin**/**LEF1 signaling activity by targeting transcription factor E2F1. E2F1 transcriptionally activates β-catenin-interacting protein 1 gene (CTNNBIP1) leading to the inhibition of β-catenin activity, and furthermore, E2F1 can induce axin2 expression and causes β-catenin degradation. TBE: TCF/LEF binding element; MBE: c-MYC proto-oncogene (MYC) binding element.

Auto-regulatory feedback loops play a vital role in the regulation of gene expression, especially in cooperation with miRNAs, which aid in balancing complex regulatory networks (Zhou et al., 2011). The auto-regulatory loop between the miR-17-92 cluster and the E2F family has been described in neural stem cells, myoblasts, Hela cells, and palatal mesenchymal cells (Sylvestre et al., 2007; Palm et al., 2013; Luo et al., 2016; Li et al., 2017). The canonical Wnt/β-catenin pathway is activated when the Wnt ligand binds to the Frizzled (FZD) receptor and its co-receptor from the low-density lipoprotein receptor-related protein 5/6 class (LRP5/6) (MacDonald et al., 2009). Previous studies have demonstrated that the miR-17-92 cluster could regulate Wnt signaling via directly targeting Wnt pathway components, such as FZD4 and LRP6 (Landskroner-Eiger et al., 2015) or the Wnt signaling inhibitors FRZB and HIPK1 (Wu et al., 2013; Spagnuolo et al., 2019). In addition, the miR-17-92 cluster could inhibit Wnt signaling via targeting E2F1. E2F1 transcriptionally activates β-catenin-interacting protein 1 gene (CTNNBIP1) and inhibits β-catenin activity (Wu et al., 2011). Moreover, E2F1 induces axin2 and causes β-catenin degradation (Hughes and Brady, 2006). In the present study, we demonstrated that the Wnt signaling pathway directly regulates miR-17-92 cluster transcription via its two key mediators β-catenin and LEF1. Previous studies have shown that c-Myc and cyclin D, two Wnt downstream targets (He et al., 1998; Shtutman et al., 1999), directly activate the transcription of MIR17HG (O’donnell et al., 2005; Yu et al., 2008; Kumar et al., 2013). Taken together, these data suggest that the miR-17-92 cluster and the Wnt/β-catenin signaling pathway form a complex auto-regulatory feedback loop in regulating cellular processes (Figure 6).

In the present study, our results demonstrated that low doses of LEF1 increased MIR17HG promoter activity, whereas higher doses of LEF1 exhibited the opposite effect (Figure 1). Consistent with our results, a previous study showed that low LEF1 concentrations activated and high LEF1 concentrations inhibited transcription of its target gene, cyclin D1 (Shtutman et al., 1999). It is possible that at high doses, LEF1 acts as a transcriptional repressor by recruiting Groucho (Gro) proteins (Eastman and Grosschedl, 1999). TCF/LEF factors have previously been shown to interact with Gro proteins and inhibit target promoter activity (Roose et al., 1998).

In the present study, 3 mM LiCl resulted in increased MIR17HG promoter activity and endogenous expression of the miR-17-92 cluster, while 30 mM LiCl led to decreased MIR17HG promoter activity and the endogenous expression of the miR-17-92 cluster. Similarly, it has been shown that low concentrations of LiCl promoted, and high concentrations inhibited, Six2 expression (Liu et al., 2016). It has been known that MIR17HG is regulated by multiple transcription factors (Fuziwara and Kimura, 2015) and signaling pathways (Trenkmann et al., 2013), The opposite effects of low and high concentration of LiCl on MIR17HG promoter activity and the endogenous expression of the miR-17-92 cluster may due to the presence of different transcription factors and active signaling pathways in the cells treated with LiCl at low and high concentrations. For example, at low concentration of LiCl the Wnt/β-catenin signaling pathway is activated (Klein and Melton, 1996), whereas, although at high concentration of LiCl (20 mM), the Wnt/β-catenin signaling pathway was also activated, the NF-κB signaling pathway is inhibited (Hoeflich et al., 2000).

The Wnt/β-catenin signaling pathway is involved in regulating the proliferation of both normal and cancer cells (Teo and Kahn, 2010; Valkenburg et al., 2011; Holland et al., 2013). In the present study, we demonstrated that 3 mM LiCl activated the Wnt/β-catenin signaling pathway, upregulated miR-17-92 cluster expression, and promoted the cell proliferation, whereas 30 mM LiCl downregulated miR-17-92 cluster expression, and decreased the cell proliferation (Figure 4, 5). Our previous study and others have demonstrated that overexpression of miR-17-92 cluster promotes cell proliferation (Chen et al., 2013; Wu et al., 2013; Zhang et al., 2017). Collectively, these data suggest that the Wnt/β-catenin pathway regulates cell proliferation at least in part via upregulation of the miR-17-92 cluster.

Interestingly, the present study demonstrated that low (3 mM) and high (30 mM) doses of LiCl exhibited opposite effects on cell proliferation (Figure 5). A previous study also demonstrated that a low dose of LiCl (4 mM) promoted the proliferation of human mesenchymal stem cells, while higher doses of LiCl (20 or 40 mM) inhibited cell proliferation (De Boer et al., 2004). It is presumed that LiCl-mediated inhibition of cell proliferation at high doses occurs in a Wnt-independent manner (De Boer et al., 2004). Low doses of LiCl were able to activate the Wnt/β-catenin pathway and stimulated the expression of multiple cell cycle regulators, including cyclins D1 and D2, resulting in enhanced pancreatic β cell proliferation (Rulifson et al., 2007). However, high doses of LiCl downregulated PKB/Akt and cyclin E, which are required for cell survival and proliferation, resulting in inhibition of hepatocellular carcinoma cell growth (Erdal et al., 2005).

Wnt signaling is one of the key signaling pathways regulating development and tightly linked to cancer. LiCl is a well-established inhibitor of Glycogen synthase kinase-3 (GSK3) and widely used to activate Wnt/β-catenin signaling in development and cancer studies (Teo and Kahn, 2010; Valkenburg et al., 2011; Holland et al., 2013). LiCl treatment can induce apoptosis in cancer cells (Kaufmann, 2011) and reduce the incidence of cancer of non-epithelial origin (Cohen et al., 1998). In addition, it has been shown that LiCl treatment can inhibit cancer cell proliferation through Wnt signaling (Qi et al., 2006). In the present study, our results showed that LiCl treatment affects the expression of miR-17-92 cluster, which is known as oncomir-1 and plays vital roles in a variety of cellular processes such as cell proliferation and apoptosis. Our finding may contribute to a better understanding of the precise mechanisms behind the action of LiCl on cancer cells.

In conclusion, our results demonstrated that miR-17-92 cluster is a target of the Wnt/β-catenin/LEF1 pathway and that Wnt/β-catenin/LEF1 signaling pathway promotes cell proliferation at least in part via direct upregulation of miR-17-92 cluster.

## Author Contributions

NW conceived and supervised the study. NW, FM, and TX designed the experiments. FM, JH, and TX performed the experiments. FM wrote the manuscript. All authors made manuscript revisions.

## Conflict of Interest Statement

The authors declare that the research was conducted in the absence of any commercial or financial relationships that could be construed as a potential conflict of interest.
