# Supplementary material for: The Wnt/β-Catenin/LEF1 Pathway Promotes Cell Proliferation at Least in Part Through Direct Upregulation of miR-17-92 Cluster
Source: Front Genet. 2019 May 29;10:525. doi: 10.3389/fgene.2019.00525 (PMC6549003; doi:10.3389/fgene.2019.00525)
Supplement: Supplementary file 1 [file Table_1.docx]

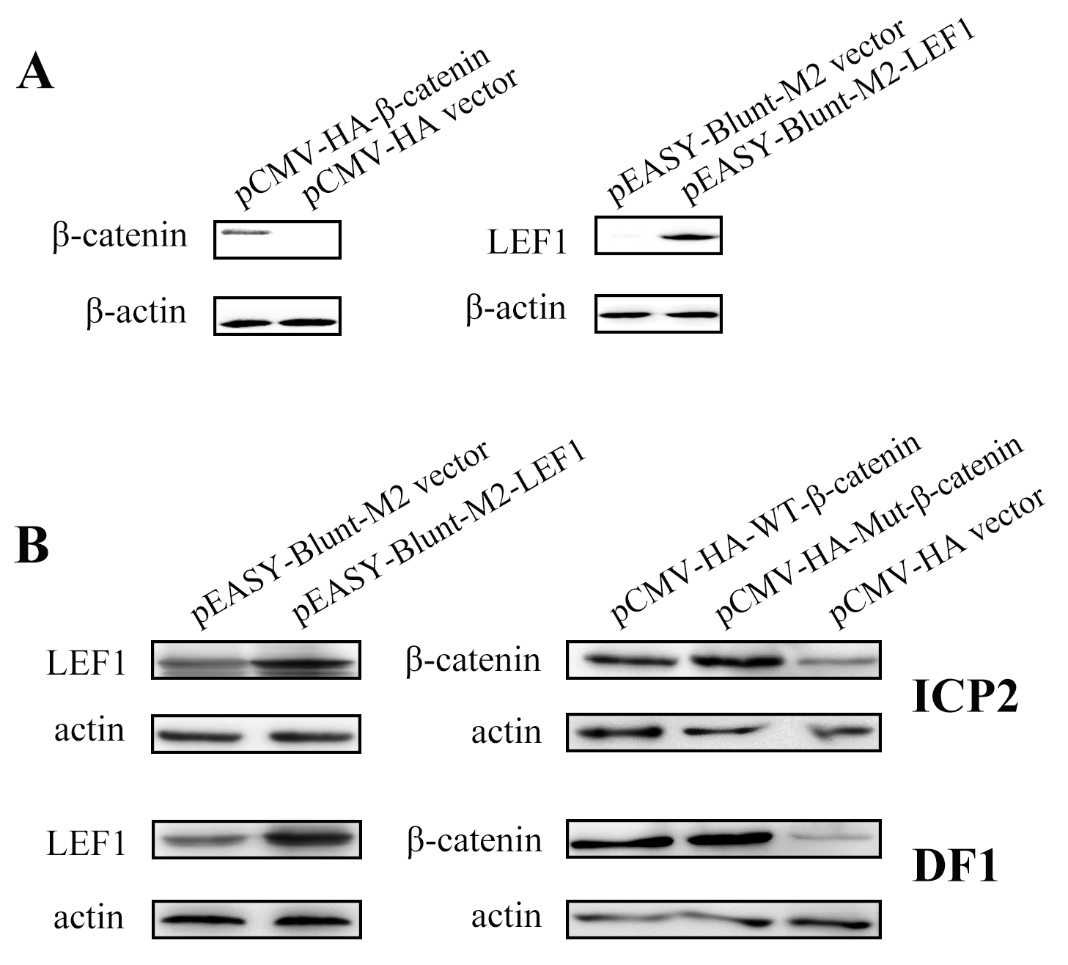


Supplementary Figure S1 Western blot identification of β-catenin and LEF1 expression vectors in ICP2 cells. Cells were transfected with pCMV-HA-β-catenin, pEASY-Blunt-M2-LEF1, pCMV-HA, and pEASY-Blunt-M2, respectively. Cells were harvested, and β-catenin and LEF1 expression were verified by western blotting using anti-HA (A), anti-Myc (A), anti-LEF1 (B) and anti-β-catenin antibodies (B), respectively. β-actin was used as a loading control.


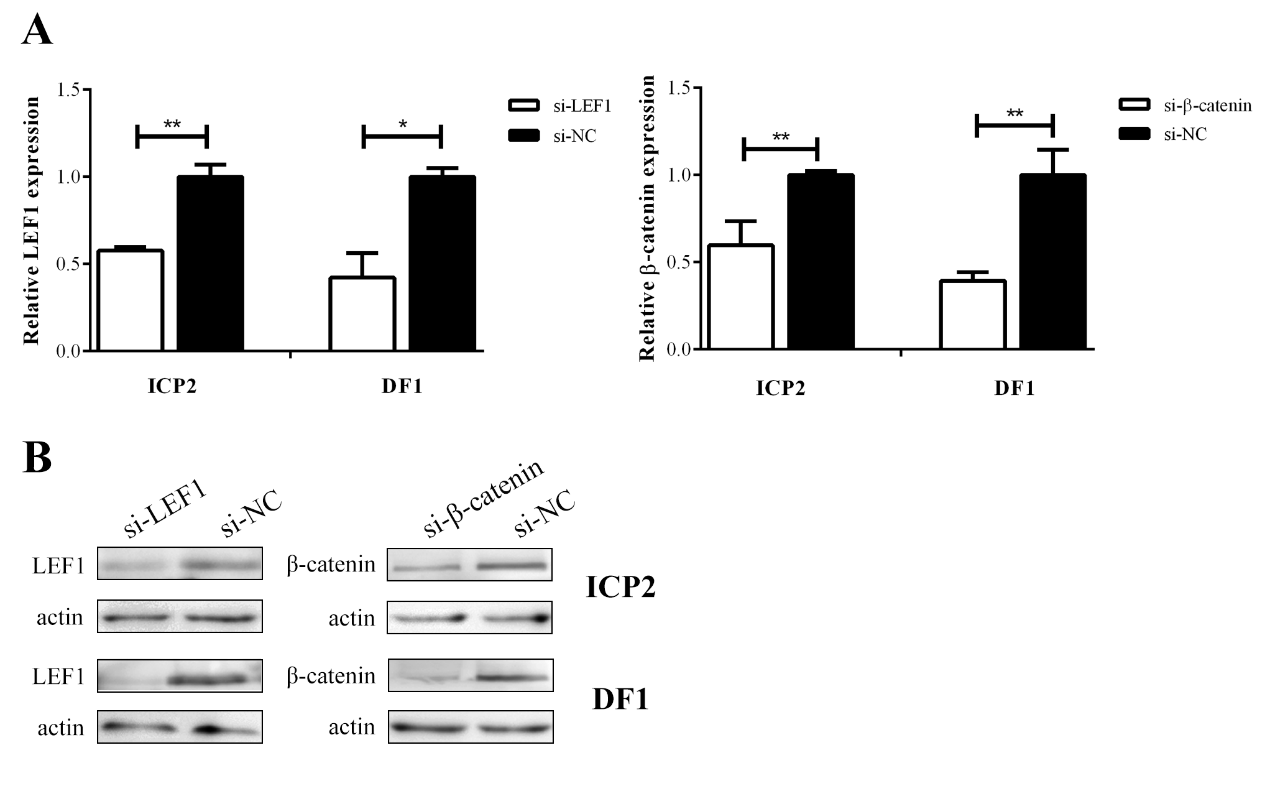


Supplementary Figure S2 Quantitative RT-PCR (A) and Western blot analysis (B) showing the knockdown efficiency of LEF1 and β-catenin by small interfering RNA in DF1 and ICP2 cells. Cells were transfected with either si-β-catenin, si-LEF1 or si-NC by Lipofectamine 2000. The knockdown efficiency of β-catenin and LEF1 at the mRNA level was verified by real-time RT-PCR. The relative gene expression was normalized to TBP. (A). The knockdown efficiency of β-catenin and LEF1 at the protein level was verified by western blotting using antibodies against LEF1 and β-catenin. β-actin served as the loading control. All data are representative of three independent experiments and shown as the mean ± SD. **P* < 0.05; ***P* < 0.01; determined by two-tailed Student’s t-test.


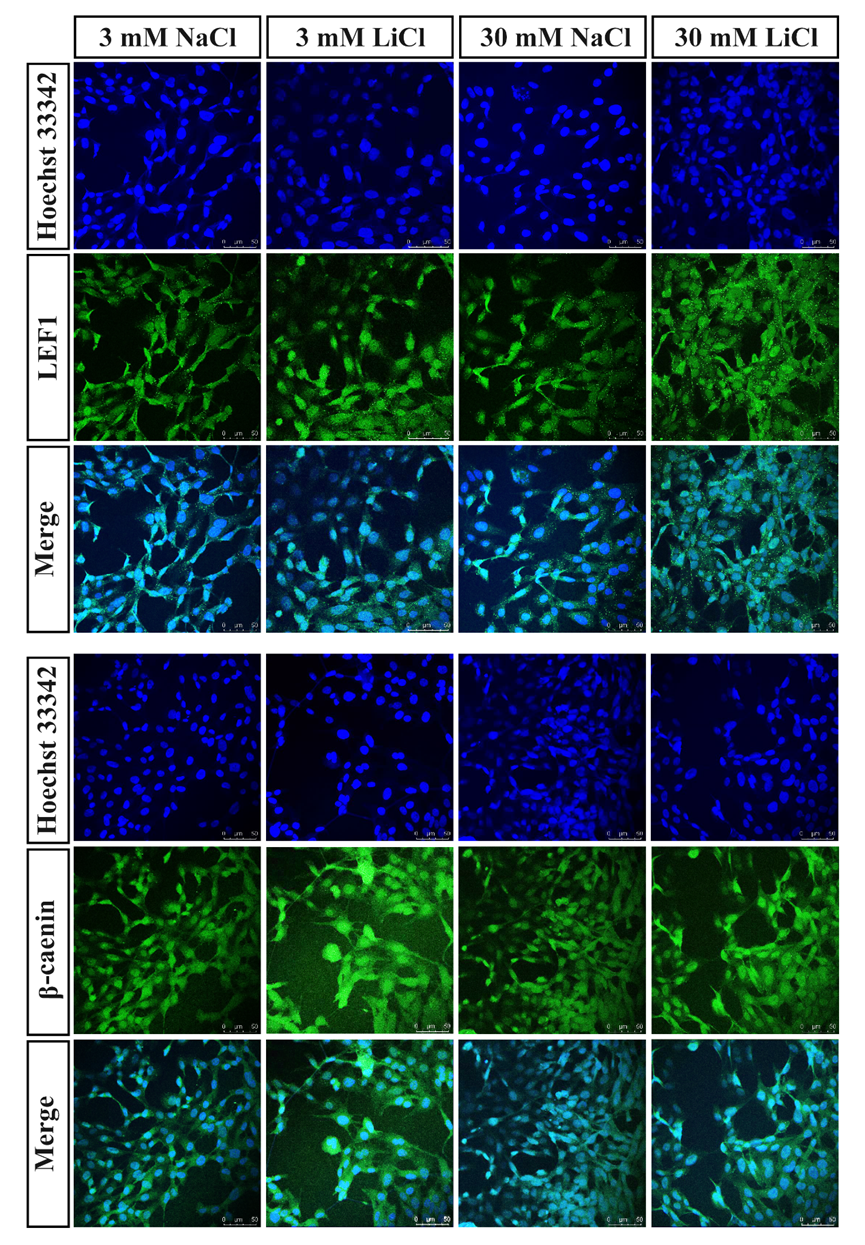


Supplementary Figure S3 Immunofluorescence showing nuclear levels of β-catenin and LEF1 in DF1 cells treated at indicated concentrations of LiCl.
